# Sexually Transmitted Bedfellows: Exquisite Association Between HIV and Herpes Simplex Virus Type 2 in 21 Communities in Southern Africa in the HIV Prevention Trials Network 071 (PopART) Study

**DOI:** 10.1093/infdis/jiy178

**Published:** 2018-04-06

**Authors:** John Bradley, Sian Floyd, Estelle Piwowar-Manning, Oliver Laeyendecker, Alicia Young, Nomtha Bell-Mandla, Justin Bwalya, Peter Bock, Sarah Fidler, Helen Ayles, Richard J Hayes, HPTN 071 (PopART) Study Team

**Affiliations:** 1 MRC Tropical Epidemiology Group, United Kingdom; 2 Department of Clinical Research, London School of Hygiene and Tropical Medicine, United Kingdom; 3 HIV Clinical Trials Unit, Department of Medicine Imperial College London, United Kingdom; 4 HIV Prevention Trials Network Laboratory Center, Johns Hopkins University School of Medicine, Baltimore, Maryland; 5 Laboratory of Immunoregulation, National Institute of Allergy and Infectious Diseases, National Institutes of Health, Baltimore, Maryland; 6 Vaccine and Infectious Disease Division, Fred Hutchinson Cancer Research Center, Seattle, Washington, USA; 7 Desmond Tutu TB Centre, Department of Paediatrics and Child Health, Stellenbosch University, Parow, South Africa; 8 Zambart, University of Zambia School of Medicine, Lusaka

**Keywords:** HIV, HSV2, South Africa, Zambia, cofactor, urban, ecological analysis

## Abstract

**Background:**

Human immunodeficiency virus (HIV) and herpes simplex virus type 2 (HSV2) are strongly associated, although mechanisms are not fully understood. An HIV prevention trial allowed reexamination of this association at individual and community levels.

**Methods:**

The HIV Prevention Trials Network 071 (PopART) study evaluates a combination prevention intervention in 21 urban communities in Zambia and South Africa. To measure impact on HIV infection incidence, a cohort of approximately 2000 adults (age range, 18–44 years) was selected randomly from each community. Baseline data on sociodemographic characteristics, behavior, and HIV/HSV2 serologic findings were used to examine the association between HIV and HSV2. At the community level, HIV prevalence was plotted against HSV2 prevalence.

**Results:**

A total of 38691 adults participated. HSV2 prevalence among women and men was 50% and 22%, respectively, in Zambia and 60% and 27%, respectively, in South Africa. Estimated HSV2 infection incidence among those aged 18–24 years was 8.06 cases/100 person-years (95% confidence interval [CI], 6.76–9.35) and 1.76 cases/100 person-years (95% CI, 1.30–2.22) among women and men, respectively. A 6-fold higher odds of HIV infection was seen in HSV2-infected individuals in both sexes, after adjustment for confounders (odds ratio, 6.66 [95% CI, 6.07–7.31] among women and 6.57 [95% CI, 5.56–7.77] among men). At the community-level, there was a strong linear relationship between HIV and HSV2 prevalence (ρ = 0.92; *P* < .001).

**Conclusions:**

There was an exquisite association between these 2 infections, at the individual and community levels, likely due in part to a powerful cofactor effect of HSV2 on HIV transmission. HSV2 control could contribute to HIV prevention.

## Teaser

Observational data from 21 communities in Southern Africa show an extremely strong relationship between herpes simplex virus type 2 (HSV2) and human immunodeficiency virus (HIV) at the individual and ecological levels. If this reflects a causal effect of HSV2 on HIV transmission, HSV2 control may contribute to HIV prevention.

Herpes simplex virus type 2 (HSV2) is the main cause of genital herpes and the most common sexually transmitted virus worldwide, with an estimated 417 million infections in adults aged 15–49 years [1]. Overall estimates of the prevalence of infection in different populations are difficult to compare and interpret because HSV2 is a lifelong incurable infection and has a prevalence that increases with age. Nevertheless, it is clear that prevalence varies substantially between regions and countries, presumably reflecting varying patterns of sexual behavior that are, in turn, determined by underlying demographic, social, and economic factors [1]. Worldwide prevalence among adults aged 15–49 years is estimated at 11%, with values of 7% in Europe, 14% in the Americas, and 32% in Africa [1]. Studies in sub-Saharan Africa have shown that the HSV2 prevalence is low before the age of sexual debut; increases rapidly during adolescence and early adulthood, with a higher prevalence among young women than among young men; and plateaus at 70%–80% in both sexes after 30 years of age [2–4].

A remarkably consistent finding has been the close epidemiological association between HSV2 and HIV. A meta-analysis of longitudinal studies showed a 3-fold increase in the risk of HIV acquisition in individuals with HSV2 infection [5], and there is evidence of a stronger cofactor effect in those in the early, more symptomatic phases of HSV2 infection [6–9]. Moreover, HSV2 in individuals who are already infected with HIV seems to increase the shedding of HIV, possibly resulting in a greater risk of HIV transmission to sex partners [10, 11]. Modeling studies have shown that HSV2 may have played an important role in facilitating the spread of HIV in sub-Saharan Africa [12, 13], and an ecological study in 4 cities with contrasting HIV epidemics identified differences in HSV2 prevalence as one of the main factors that might help to explain differences in HIV prevalence [4, 14, 15].

There is a strong biological rationale for a facilitating effect of HSV2 on HIV transmission, owing to the episodic genital lesions associated with herpes, as well as subtler effects at the cellular level that may persist while HSV2 is asymptomatic [16]. Moreover, HSV2 infection results in an increase in the density of target cells for HIV in the male foreskin [17]. However, a series of randomized trials of acyclovir treatment in those with HSV2 infection found no evidence of an effect on HIV acquisition or transmission, probably because the treatment provided was insufficient to control the expression of HSV2 and avert its potentiating effect on HIV infection [18–20]. Work is now in progress on the development and evaluation of HSV2 vaccines, motivated both by a need to reduce the burden of ill health associated with genital herpes and to establish a potential strategy to prevent HIV acquisition [21–24].

A large community-randomized trial of an HIV combination prevention intervention offered an opportunity to reevaluate the association between HIV and HSV2 in large urban and peri-urban settlements in Southern Africa. The HIV Prevention Trials Network (HPTN) 071 (PopART) study is evaluating the impact of an intervention involving universal testing and treatment on the incidence of HIV infection at the population level in 21 communities in Zambia and South Africa [25]. We have used baseline data from the nearly 40000 adults in this trial, to measure the association between the 2 infections at individual level, and also in an ecological analysis at the community level.

## METHODS

### Study Design

Full details of the HPTN 071 (PopART) trial have been presented previously [25]. In brief, 21 urban and peri-urban communities in Zambia and South Africa (each community was defined as the catchment population of a health facility) have been randomly allocated to 3 study arms. Arm A is receiving the full PopART intervention, which comprises universal annual home-based HIV testing and counseling provided by lay community health workers, who also assist with referral, linkage to care, and adherence support for clients who need HIV-related services, in addition to the offer of immediate initiation of antiretroviral therapy (ART) irrespective of CD4^+^ T-cell count at the local clinic. Arm B is receiving the full PopART intervention except that ART is provided according to current national treatment guidelines. Arm C is a control arm that continues to receive current national standard of care. During 2016, all arm B and C clinics transitioned to providing ART irrespective of CD4^+^ T-cell count, in accordance with changing national guidelines.

The impact of the PopART intervention on HIV infection incidence is being measured in a population cohort comprising a random sample of adults from the general population, who will be followed up annually for 36 months. A brief census was performed in each community prior to the trial and was used to select a random sample of households. With the agreement of the household head, all adults aged 18–44 years residing in each selected household were enumerated, and one was selected at random for enrollment in the population cohort, with an initial target of 2500 adults in each community. Data for the analysis presented in this article came from the baseline survey of the population cohort, which was performed between November 2013 and March 2015.

Following informed consent, data on sociodemographic, behavioral, and other variables were collected from population cohort participants, using an interviewer-administered questionnaire on a handheld electronic-data-capture device. At the end of the interview, a blood sample was collected by a trained research nurse and transported to the laboratory for processing, storage, and analysis. The participant was informed that testing would be performed at a later date for research purposes and that results would not be returned, but for those wishing to know their HIV status the nurse offered an on-the-spot rapid HIV test, using a finger-prick blood sample.

### Laboratory Methods

HIV and HSV2 status were determined by testing blood samples collected from consenting survey participants. To determine HIV status, blood samples underwent in-country analysis by a single fourth-generation assay (Architect HIV Ag/Ab Combo Assay, Abbott Diagnostics, Delkenheim, Germany). Further testing was performed at the HPTN Laboratory Center (Baltimore, MD). Samples that had reactive results of in-country analysis were tested with a second fourth-generation assay (GS HIV Combo Assay, Bio-Rad Laboratories, Redmond, WA). Samples with discrepant/discordant test results were tested with additional assays to determine HIV status. For HSV2 status, blood samples underwent in-country analysis by the Kalon HSV2 immunoglobulin G enzyme-linked immunosorbent assay (ELISA; Kalon Biological, United Kingdom), where the in-country laboratories’ performance was validated by the HPTN laboratory core [26]. The manufacturer’s calibrator was run in duplicate on all plate runs in the study and OD units from the ELISA plates were converted to index values. The manufacturer’s index value cutoffs (<0.9, negative; 0.9–1.1, indeterminate; and >1.1, positive) were used to assign sample results. For the current study, samples with an indeterminate result were considered negative for HSV2.

### Statistical Methods

Data from the baseline survey of the population cohort were first used to present the overall prevalence of HSV2 by age and sex in each country. The incidence of HSV2 infection in the group aged 18–24 years was estimated from the age-specific seroprevalence, assuming a constant rate of seroconversion. The probability of being seropositive at age *A* was assumed to be *k* + [1 − *k*][1 − *e*^−λ(*A* − 18)^], where λ is the seroconversion rate and *k* is the seroprevalence at age 18 years. To account for clustering, a community-level, normally distributed random effect was added on the logit scale. Estimation was performed using the maximum likelihood method.

Next we performed logistic regression analysis to explore risk factors for HSV2 infection at the individual level, stratified by sex, with adjustment for age group and study community. A hierarchical approach was used for model fitting [27], with the effects of sociodemographic variables adjusted for other sociodemographic variables and the effects of behavioral and biological variables adjusted for all other variables. We tested for interactions between country and each variable to assess whether associations were similar in Zambia and South Africa. We then examined the association between HSV2 and HIV infection at the individual level, adjusting for age group, sex, study community, and all other variables that were confounding factors for this association.

Finally, we performed an ecological analysis to explore the association between HIV prevalence and HSV2 prevalence at the population level. We plotted the community-level HIV prevalence against the community-level HSV2 prevalence. We also examined whether differences in HSV2 prevalence across communities could be explained on the basis of measured behavioral or other variables.

### Ethical Considerations

All participants in the population cohort gave written informed consent. The study and all of the procedures described above were approved by the ethics committees of the London School of Hygiene and Tropical Medicine, the University of Zambia, and Stellenbosch University.

## RESULTS

A total of 38691 adults aged 18–44 years were enrolled in the population cohort during the baseline survey (19750 in Zambia and 18941 in South Africa), which was 74% of the target number of 52500. Numbers enrolled per community varied from 1007 to 2559 individuals. A complete questionnaire was completed and a blood specimen was collected for 36954 participants (96%). Laboratory results for HSV2 infection and confirmed HIV status were available for 37284 participants (96%) and 37334 participants (96%), respectively.

### HSV2 Prevalence by Age, Sex, and Country

**Figure 1.**
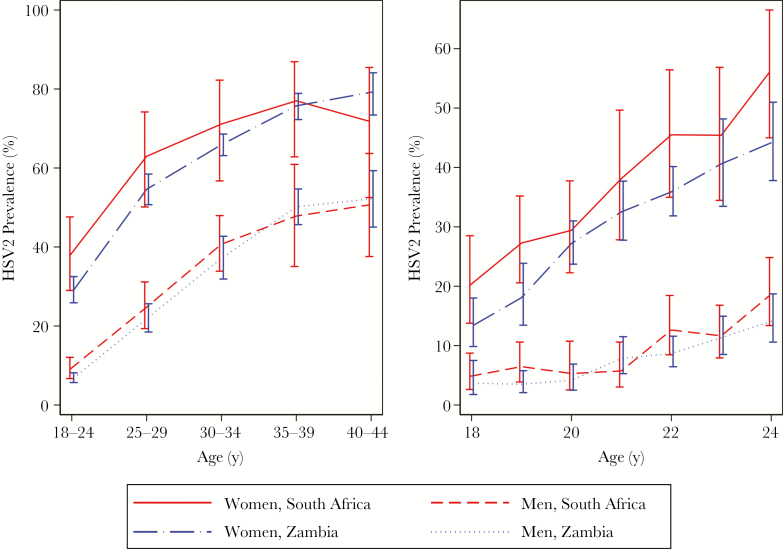
Herpes simplex virus type 2 (HSV2) prevalence, by sex, country, and age group, in all age groups (left) and those aged <25 years (right).

The overall prevalence of HSV2 infection among women and men was 50% and 22%, respectively, in the 12 Zambian communities and 60% and 27%, respectively, in the 9 South African communities. Prevalence by age, sex, and country is shown in Figure 1 and Supplementary Table 1. Prevalence increased steeply with age and was consistently higher in women than men in every age group in both countries. HSV2 prevalence was already very high in women aged 18–24 years (29% in Zambia and 38% in South Africa), increasing to over 70% by age 35 years. In men, HSV2 prevalence was much lower in the youngest age group (7% in Zambia and 9% in South Africa), increasing to around 50% in men aged ≥35 years. Figure 1 also shows the prevalence by year of age among men and women aged 18–24 years. Around 20% of 18-year-old women in South Africa were infected with HSV2, compared with 13% in Zambia, but in both countries the prevalence increased to >40% by age 24 years. Prevalence was much lower in men aged 18 years (5% in South Africa and 4% in Zambia). The incidence in the group aged 18–24 years was estimated to be 8.06 cases/100 person-years (95% confidence interval [CI], 6.76–9.35) and 1.76 cases/100 person-years (95% CI, 1.30–2.22) among women and men, respectively.

### Risk Factors for HSV2 Infection

Table 1 shows associations between sociodemographic and behavioral/biological variables and HSV2 infection separately for men and women (data are from both countries combined). After adjustment for age, community, and other factors, associations with several of the variables examined were observed in both sexes. HSV2 prevalence increased with lower levels of education, more nights spent away from home, and lower socioeconomic status and was higher among those who were widowed, divorced, or separated. It also increased steeply with reported lifetime number of sex partners and was higher among those with a high Audit score, indicating possible alcohol dependence. Among women but not men, the HSV2 prevalence increased among those with a younger age at first sex and those who reported use of condoms at last sex. There were also associations with ethnic group, but a large number of ethnic groups were represented, and these associations were quite strongly confounded with study community. These effects of ethnic group are not presented or discussed further.

**Table 1.** Risk Factors for Herpes Simplex Virus Type 2 (HSV2) Infection Among Women and Men in 21 Communities in Zambia and South Africa

Variable	Women					Men				
	HSV2 Positivity, Proportion (%)	OR^a^ (95% CI)	*P*	aOR^b^ (95% CI)	*P*	HSV2 Positivity, Proportion (%)	OR^a^ (95% CI)	*P*	aOR^b^ (95% CI)	*P*
Sociodemographic										
Age, y			<.001		<.001			<.001		<.001
18–24	3229/9929 (33)	1		1		372/4775 (8)	1		1	
25–29	3406/5812 (59)	2.97 (2.78–3.18)		2.87 (2.67–3.10)		504/2148 (23)	3.52 (3.04–4.08)		3.19 (2.74–3.73)	
30–34	3187/4652 (69)	4.71 (4.35–5.09)		4.20 (3.86–4.58)		628/1605 (39)	7.54 (6.51–8.74)		5.96 (5.06–7.03)	
35–39	2548/3335 (76)	7.02 (6.39–7.71)		6.21 (5.60–6.88)		599/1225 (49)	11.34 (9.70–13.26)		8.94 (7.48–10.69)	
40–44	1835/2449 (75)	6.87 (6.18–7.64)		5.92 (5.26–6.66)		527/1027 (51)	12.63 (10.71–14.90)		9.78 (8.06–11.86)	
Education level			<.001		<.001			<.001		.006
None/grade 1–2	349/571 (61)	1		1		44/112 (39)	1		1	
Grade 3–6	1329/2120 (63)	1.17 (.96–1.44)		1.19 (.97–1.47)		190/530 (36)	0.86 (.55–1.36)		0.84 (.53–1.33)	
Grade 7–10	6561/11540 (57)	1.00 (.83–1.21)		1.05 (.87–1.27)		1104/4128 (27)	0.92 (.60–1.39)		0.92 (.60–1.42)	
Grade 11–12	5277/10417 (51)	0.67 (.55–.81)		0.76 (.62–.92)		1090/5101 (21)	0.78 (.51–1.19)		0.81 (.53–1.24)	
College/university	575/1319 (44)	0.48 (.38–.60)		0.58 (.46–.73)		158/766 (21)	0.59 (.37–.93)		0.63 (.39–1.01)	
Marital status			<.001		<.001			<.001		<.001
Married	6980/12185 (57)	1		1		1075/2679 (40)	1		1	
Never married	5468/11636 (47)	0.86 (.80–.92)		0.92 (.86–.99)		1291/7519 (17)	0.71 (.62–.81)		0.69 (.61–.79)	
Divorced/separated	1252/1729 (72)	2.04 (1.81–2.29)		1.92 (1.70–2.17)		218/484 (45)	1.23 (1.00–1.51)		1.18 (.95–1.46)	
Widowed	463/540 (86)	2.95 (2.29–3.79)		2.82 (2.18–3.66)		34/57 (60)	1.57 (.91–2.72)		1.55 (.89–2.71)	
Nights away from home in past 3 mo, no.			<.001		<.001			.036		.009
0	12043/22243 (54)	1		1		2203/8988 (25)	1		1	
1–6	1028/1794 (57)	1.16 (1.04–1.29)		1.21 (1.08–1.35)		180/775 (23)	1.19 (.98–1.46)		1.24 (1.01–1.51)	
≥7	743/1248 (60)	1.22 (1.07–1.39)		1.27 (1.11–1.45)		182/638 (29)	1.24 (1.01–1.53)		1.30 (1.06–1.60)	
SES quartile			<.001		<.001			<.001		.001
1 (lowest)	4126/6703 (62)	1		1		798/2528 (32)	1		1	
2	3815/6509 (59)	0.82 (.75–.89)		0.86 (.79–.94)		739/2621 (28)	0.94 (.81–1.07)		0.94 (.82–1.09)	
3	3422/6649 (52)	0.67 (.61–.72)		0.73 (.67–.80)		598/2723 (22)	0.81 (.70–.94)		0.83 (.71–.97)	
4 (highest)	2700/6056 (45)	0.52 (.47–.57)		0.62 (.56–.69)		469/2808 (17)	0.68 (.58–.80)		0.71 (.60–.85)	
Behavioral/biological										
Age at first sex, y			<.001		.003			.357		.774
≤15	2153/3558 (61)	1		1		583/2279 (26)	1		1	
16–18	7056/12346 (57)	0.86 (.79–.94)		1.04 (.94–1.15)		1001/3948 (25)	0.97 (.85–1.10)		0.99 (.84–1.18)	
≥19	3575/6660 (54)	0.62 (.57–.68)		0.90 (.80–1.01)		623/2238 (28)	0.90 (.77–1.05)		1.06 (.86–1.29)	
Sex partners, lifetime no.			<.001		<.001			<.001		<.001
0	244/1661 (15)	1		1		99/1259 (8)	1		1	
1	3815/8978 (42)	2.51 (2.16–2.92)		2.69 (2.19–3.30)		465/2233 (21)	1.43 (1.11–1.83)		1.65 (1.18–2.29)	
2–4	6424/10314 (62)	5.33 (4.58–6.21)		5.52 (4.49–6.79)		719/3121 (23)	1.57 (1.23–2.01)		1.66 (1.20–2.29)	
5–9	1608/2065 (78)	9.35 (7.77–11.26)		9.33 (7.34–11.85)		491/1623 (30)	2.02 (1.56–2.61)		2.21 (1.57–3.12)	
≥10	356/419 (85)	13.02 (9.48–17.89)		11.41 (7.85–16.57)		415/1101 (38)	2.55 (1.95–3.34)		2.97 (2.07–4.27)	
Sex partners in past 12 mo, no.			<.001					<.001		
0	3000/6653 (45)	1		…		590/3645 (16)	1		…	
1	10078/17727 (57)	1.30 (1.22–1.39)		…		1518/5160 (29)	1.37 (1.21–1.55)		…	
2–3	510/730 (70)	2.40 (2.01–2.88)		…		264/1020 (26)	1.46 (1.21–1.76)		…	
≥4	60/73 (82)	5.29 (2.78–10.05)		…		100/358 (28)	1.74 (1.32–2.30)		…	
Condom use during last sex			<.001		<.001			.554		.864
No	5987/11316 (53)	1		1		982/3344 (29)	1		1	
Yes	4747/7266 (65)	1.35 (1.26–1.45)		1.21 (1.11–1.31)		1018/4603 (23)	1.04 (.92–1.18)		1.01 (.87–1.17)	
Audit score (alcohol dependence)			<.001		.001			<.001		.012
0–7 (lower risk)	11245/24085 (53)	1		1		1865/8290 (23)	1		1	
8–15 (increasing risk)	694/1047 (66)	1.71 (1.49–1.98)		1.23 (1.03–1.46)		428/1376 (31)	1.38 (1.20–1.59)		1.31 (1.09–1.57)	
16–19 (higher risk)	132/186 (71)	2.19 (1.54–3.11)		1.67 (1.08–2.58)		87/286 (30)	1.38 (1.04–1.83)		1.27 (.97–1.94)	
≥20 (possible dependence)	125/155 (81)	3.14 (2.04–4.83)		1.98 (1.17–3.33)		128/376 (34)	1.38 (1.08–1.77)		1.37 (.93–1.77)	
Ever taken recreational drugs			.027		.462			.702		.010
No	13731/25274 (54)	1		1		2238/9047 (25)	1		1	
Yes	386/763 (51)	1.21 (1.02–1.42)		1.08 (.88–1.33)		374/1656 (23)	0.97 (.84–1.12)		0.78 (.64–.94)	
Circumcision status								<.001		<.001
Not circumcised	…	…		…		1169/5474 (21)	1		1	
VMMC	…	…		…		293/1811 (16)	0.85 (.72–.99)		0.94 (.77–1.14)	
TMC	…	…		…		1079/3102 (35)	1.55 (1.33–1.82)		1.49 (1.21–1.83)	

Abbreviations: aOR, adjusted odds ratio; CI, confidence interval; OR, odds ratio; SES, socioeconomic status; TMC, traditional male circumcision; VMMC, voluntary medical male circumcision.

^a^Adjusted for age group and community.

^b^Sociodemographic variables are adjusted for age group, community, and all other sociodemographic variables in this table. Behavioral/biological variables are adjusted for age group, community, and all other sociodemographic and behavioral/biological variables in this table, apart from the number of sex partners in the previous 12 months, which was omitted because of collinearity.

The association of 3 variables with HSV2 differed significantly by country (Supplementary Tables 2 and 3). In South Africa, there was evidence that HSV2 infection was more prevalent among men who had undergone traditional circumcision than among those who were uncircumcised or who had undergone medical circumcision, but this effect was not seen in Zambia (test for interaction: *P* = .004), while in Zambia but not in South Africa, HSV2 infection was more prevalent among men with lower levels of education (test for interaction: *P* < .001). Effects of marital status varied between countries in both sexes. In Zambia, men and women who were single had a decreased risk of HSV2 infection, while South African women who were single had an increased risk (test for interaction: *P* < .001).

### Association Between HSV2 and HIV at the Individual Level

The HIV prevalence in women and men was 27% and 11%, respectively, in South Africa and 25% and 13%, respectively, in Zambia. Table 2 shows the association between HSV2 and HIV at the individual level in men and women. Odds ratios (ORs) are adjusted for age and community and then additionally for other confounding factors. There was a very strong association, with a 6-fold higher odds of HIV infection among HSV2-infected individuals in both sexes, even after adjustment for other risk factors, including lifetime number of sex partners (adjusted OR, 6.66 [95% CI, 6.07–7.31] among women and 6.57 [95% CI, 5.56–7.77] among men).

**Table 2.** Association between Herpes Simplex Virus Type 2 (HSV2) Infection and Human Immunodeficiency Virus (HIV) Infection, Overall and by Country

	Women			Men		
Variable	HIV Positivity, Proportion (%)	OR^a^ (95% CI)	aOR^b^ (95% CI)	HIV Positivity, Proportion (%)	OR^a^ (95% CI)	aOR^b^ (95% CI)
Overall						
HSV2 negative	791/11970 (7)	1	1	368/8149 (5)	1	1
HSV2 positive	5986/14204 (42)	7.70 (7.08–8.37)	6.66 (6.07–7.31)	909/2629 (35)	6.84 (5.93–7.90)	6.57 (5.56–7.77)
South Africa						
HSV2 negative	346/4922 (7)	1	1	169/3996 (4)	1	1
HSV2 positive	2972/7250 (41)	6.20 (5.48–7.03)	5.49 (4.77–6.33)	444/1473 (30)	6.14 (5.00–7.53)	5.58 (4.38–7.12)
Zambia						
HSV2 negative	445/7048 (6)	1	1	199/4153 (5)	1	1
HSV2 positive	3014/6954 (43)	8.87 (7.94–9.92)	7.38 (6.52–8.36)	465/1156 (40)	5.58 (4.37–7.12)	7.66 (6.07–9.65)

Abbreviations: aOR, adjusted odds ratio; CI, confidence interval; OR, odds ratio

^a^Adjusted for age group and community.

^b^Adjusted for age group, community, lifetime number of sex partners, sexual partners in the past year, audit score, recreational drug use, education level, marital status, nights away from home in the past 3 months, socioeconomic status, and, for men, circumcision.

### Association between HSV2 and HIV at the Community Level

There was substantial variation in HSV2 prevalence between communities in both countries. This was particularly so in South Africa, where the prevalence varied from 13% to 37% among men and from 28% to 74% among women (Supplementary Table 4).

**Figure 2.**
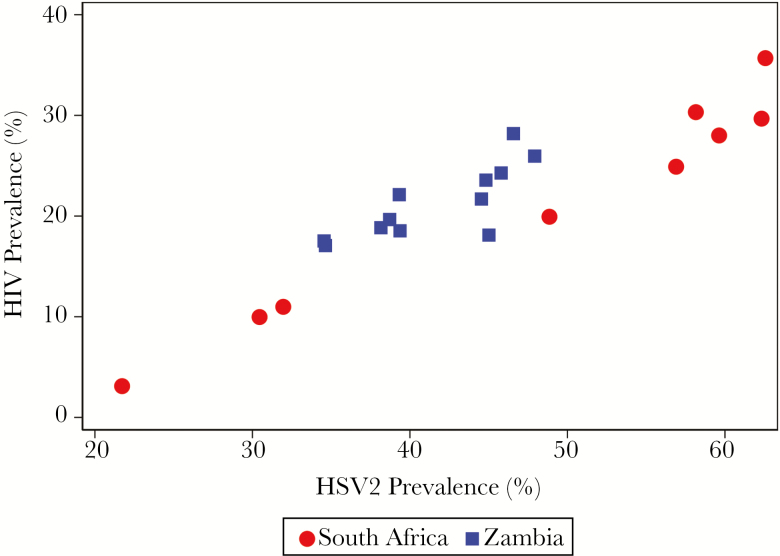
Community-level association between herpes simplex virus type 2 (HSV2) prevalence and human immunodeficiency virus (HIV) prevalence.

Figure 2 shows the association between the prevalences of HSV2 and HIV infection at the community level. There was an extremely strong linear relationship between the 2 infections, overall (ρ = 0.92; *P* < .001) and separately among men and women (Supplementary Figure 1). The overall HIV prevalence was always >25% when the HSV2 prevalence was >50%, and it was always below about 10% when the HSV2 prevalence was below about 30%.

**Figure 3.**
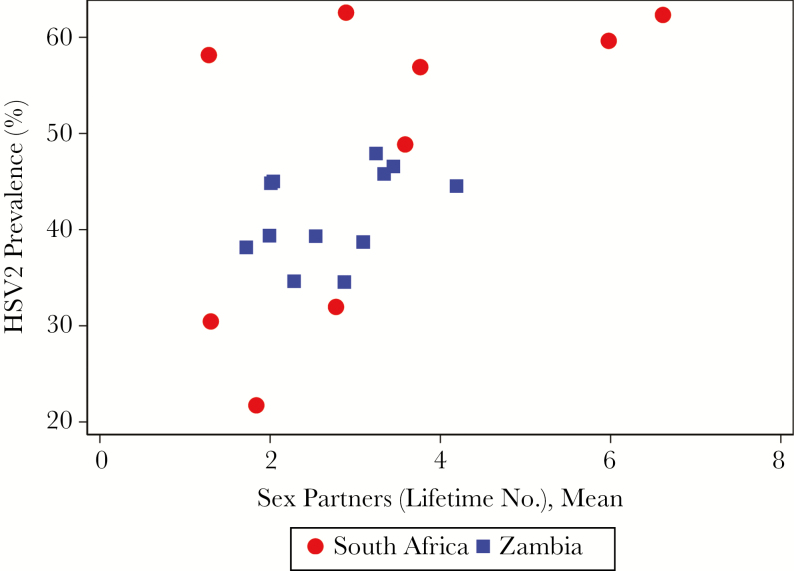
Community-level association between lifetime number of sex partners and herpes simplex virus type 2 (HSV2) prevalence.

We examined whether the variations in HSV2 prevalence could be explained by differences in reported sexual behavior. Figure 3 shows the strong association between the community HSV2 prevalence and the mean lifetime number of sex partners (ρ = 0.58; *P* = .006). However, when linear regression was used to analyze the association between HIV and HSV2 at the community level, this association remained strong after adjustment for mean number of lifetime sex partners (*P* < .001; Supplementary Table 5). The relationship between HIV prevalence and mean lifetime number of sex partners was similar and is shown in Supplementary Figure 2.

## DISCUSSION

This study of nearly 40000 men and women in 21 urban and peri-urban communities confirms the strong association between HIV and HSV2 at both the individual and community levels. At the individual level, HSV2-infected men and women had a roughly 6-fold higher odds of HIV infection than HSV2-uninfected individuals, even after allowing for other risk factors. At the community level, there was an exquisite linear relationship between the 2 infections.

Many previous studies have documented the strong association between these 2 infections at the individual level, and this finding has been remarkably consistent across different studies and settings [4, 28]. In cross-sectional studies (including the current analysis), the direction of causality cannot be definitively established, and some or all of the association might be explained by an effect of HIV on the acquisition of HSV2 infection. However, the HSV2 prevalence increases with age much more rapidly and to much higher levels than the HIV prevalence, and so it seems likely that HSV2 infection precedes HIV acquisition in most cases. Moreover, longitudinal studies have shown a strong association between prior HSV2 infection and acquisition of HIV during follow-up, consistent with a direct causal effect of HSV2 on HIV infection [5].

The association between HSV2 and HIV could be confounded by sexual risk behavior. Both infections are sexually transmitted, so HIV and HSV2 may cluster among individuals with larger numbers of sex partners. As in previous studies, we continue to find a strong association even after adjustment for age, lifetime number of sex partners, and other risk factors, but we cannot rule out residual confounding, given (1) the limitations of self-reported data on sexual behavior [5] and (2) network effects making information about lifetime number of sex partners insufficient to completely control for confounding [29].

The most definitive test of a causal effect of HSV2 on HIV acquisition would be evidence from a randomized trial of an effective intervention against HSV2 that shows a reduced incidence of HIV infection. Disappointingly, the 3 trials that have directly examined the effects of acyclovir therapy among HSV2-infected individuals who were either negative or positive for HIV showed no effect on HIV infection incidence [18–20]. However, subsequent studies have shown that the treatment regimen used for HSV2 in these trials was likely insufficiently potent to avert the strong biological interaction between the 2 infections [16].

We went on to look at the association between HSV2 and HIV at the community level. To our knowledge, the only previous ecological analysis of this kind was performed as part of the Four Cities Study that examined explanations for the different HIV epidemics in 2 cities (Cotonou, Benin, and Yaounde, Cameroon) with a relatively low HIV prevalence in West Africa and 2 high prevalence cities (Kisumu, Kenya, and Ndola, Zambia) in East and Southern Africa [4, 14]. Interestingly, this study found few differences in reported sexual behavior that could explain the different epidemics, but it identified 2 biological factors, HSV2 infection and male circumcision, that showed major differences between the cities with high and those with low prevalences [14]. However, the conclusions were limited by the small number of communities studied.

With 21 large urban and peri-urban communities, spread across 2 countries in Southern Africa with generalized HIV epidemics, the HPTN 071 (PopART) study provided much greater scope to examine this ecological correlation. The results were striking, and we were surprised by the extremely strong association between the prevalences of the 2 pathogens at the community level, such that the prevalence of HIV could be predicted almost exactly from the HSV2 prevalence. An important question is whether this relationship merely reflects variations in sexual behavior between the communities that have a common effect on the transmission dynamics of the 2 infections or whether it reflects a powerful cofactor effect of HSV2 infection on HIV acquisition and transmission.

It is likely that both mechanisms play a role in explaining the observed associations, and we are unable to quantify the importance of each. However, there are 3 reasons why we consider it likely that the biological cofactor effect of HSV2 plays an important role. First, although both viruses are sexually transmitted, they have different transmission dynamics. HSV2 is much more readily transmitted, has a much higher prevalence, is less concentrated in core groups with high rates of partner change, and has a different clinical course (a high frequency of symptomatic episodes in early infection, followed by long periods of asymptomatic infection later in life), which likely affects the pattern of transmission. It therefore seems implausible that confounding by sexual behavior would in itself produce such a strong linear relationship between the 2 infections. Second, the consistency and strength of the association between HIV and HSV2 at the individual level is unusual in HIV epidemiology and argues for a biological mechanism. Third, we now have data from detailed biological studies demonstrating the strong interaction between the viruses at mucosal and cellular levels [16].

If this conclusion is valid, it has important implications for HIV epidemiology and control. The cofactor effect of HSV2 is important not only because of its strength but because HSV2 infection is such a prevalent condition. Our study and studies by others show that a large proportion of men and women become infected with this virus by the age of 35 years in many parts of sub-Saharan Africa [1, 4]. Moreover, many individuals (especially young women) acquire their initial HSV2 infection in adolescence or early adulthood [2]. Frequent clinical episodes of genital herpes during the early years of HSV2 infection are likely to put individuals at high risk of HIV infection during the period when they are most likely to be exposed. For these reasons, mathematical models have shown that a large proportion of HIV infections may be attributable to HSV2 infection in this region [12, 13]. With the increasing recognition that a combination of preventive tools will be needed to move toward elimination of HIV as a public health problem, effective interventions against HSV2, such as the prophylactic or therapeutic vaccines currently under study, may play an important role in this struggle [21, 24].

Strengths of our study include the large number of communities studied across diverse geographical settings, the large sample of randomly selected adults from each community, and the systematic collection of data on a wide range of variables that may be risk factors for the infections under study. Limitations include the cross-sectional study design, the usual biases in self-reported data (especially on sexual risk behavior), and the potential for some bias in the study sample, given that some individuals randomly selected for inclusion could not be contacted and that men were differentially underrepresented in the sample. Both HIV and HSV2 are common in these populations, so the ORs presented here are not approximations of risk ratios.

The association between HIV and HSV2 has been known for many years but has been relatively neglected since the negative results of the acyclovir treatment trials were published [18–20]. Our data provide new and compelling evidence of the likely role of HSV2 in explaining variations in the HIV epidemic between regions and communities, as well as the very high incidence of HIV infection in some parts of sub-Saharan Africa. If this is true, renewed attention is needed to the development and evaluation of effective HSV2 control measures as tools for HIV prevention and to reduce the significant burden of disease associated with this ubiquitous virus.

## Supplementary Data

Supplementary materials are available at *The Journal of Infectious Diseases* online. Consisting of data provided by the authors to benefit the reader, the posted materials are not copyedited and are the sole responsibility of the authors, so questions or comments should be addressed to the corresponding author.

Supplementary Figure S1

Supplementary Figure S2

Supplementary Table s1

Supplementary Table s2

Supplementary Table s3

Supplementary Table s4

Supplementary Table s5

## Notes

***Acknowledgments.*** We thank all members of the HPTN 071 (PopART) Study Team and all study participants and their communities, for their contributions to this research.

***Financial support.*** This work was supported by the National Institute of Allergy and Infectious Diseases (cooperative agreements UM1-AI068619, UM1-AI068617, and UM1-AI068613HPTN 071), the US President’s Emergency Plan for AIDS Relief, the International Initiative for Impact Evaluation, the Bill and Melinda Gates Foundation, the National Institute on Drug Abuse, and the National Institute of Mental Health.

***Potential conflicts of interest.*** All authors: No reported conflicts of interest. All authors have submitted the ICMJE Form for Disclosure of Potential Conflicts of Interest. Conflicts that the editors consider relevant to the content of the manuscript have been disclosed.

Presented in part: 9th International AIDS Society Conference on HIV Science, Paris, France, 23–26 July 2017. Abstract TUPEC0756.

## References

[1] Looker KJ, Magaret AS, May MT et al Global and regional estimates of prevalent and incident herpes simplex virus type 1 infections in 2012. PLoS One 2015; 10:e0140765. 26510007 10.1371/journal.pone.0140765 PMC4624804

[2] Obasi A, Mosha F, Quigley M et al Antibody to herpes simplex virus type 2 as a marker of sexual risk behavior in rural Tanzania. J Infect Dis 1999; 179:16–24. 9841817 10.1086/314555

[3] Smith JS, Robinson NJ Age-specific prevalence of infection with herpes simplex virus types 2 and 1: a global review. J Infect Dis 2002; 186(Suppl 1):S3–28. 12353183 10.1086/343739

[4] Weiss HA, Buvé A, Robinson NJ et al; Study Group on Heterogeneity of HIV Epidemics in African Cities The epidemiology of HSV-2 infection and its association with HIV infection in four urban African populations. AIDS 2001; 15(Suppl 4):S97–108. 11686471 10.1097/00002030-200108004-00011

[5] Freeman EE, Weiss HA, Glynn JR, Cross PL, Whitworth JA, Hayes RJ Herpes simplex virus 2 infection increases HIV acquisition in men and women: systematic review and meta-analysis of longitudinal studies. AIDS 2006; 20:73–83. 16327322 10.1097/01.aids.0000198081.09337.a7

[6] del Mar Pujades Rodríguez M, Obasi A, Mosha F et al Herpes simplex virus type 2 infection increases HIV incidence: a prospective study in rural Tanzania. AIDS 2002; 16:451–62. 11834958 10.1097/00002030-200202150-00018

[7] Brown JM, Wald A, Hubbard A et al Incident and prevalent herpes simplex virus type 2 infection increases risk of HIV acquisition among women in Uganda and Zimbabwe. AIDS 2007; 21:1515–23. 17630545 10.1097/QAD.0b013e3282004929

[8] Reynolds SJ, Risbud AR, Shepherd ME et al Recent herpes simplex virus type 2 infection and the risk of human immunodeficiency virus type 1 acquisition in India. J Infect Dis 2003; 187:1513–21. 12721931 10.1086/368357

[9] Sobngwi-Tambekou J, Taljaard D, Lissouba P et al Effect of HSV-2 serostatus on acquisition of HIV by young men: results of a longitudinal study in Orange Farm, South Africa. J Infect Dis 2009; 199:958–64. 19220143 10.1086/597208 PMC2868899

[10] Todd J, Riedner G, Maboko L et al Effect of genital herpes on cervicovaginal HIV shedding in women co-infected with HIV AND HSV-2 in Tanzania. PLoS One 2013; 8:e59037. 23516595 10.1371/journal.pone.0059037 PMC3596319

[11] Johnson LF, Lewis DA The effect of genital tract infections on HIV-1 shedding in the genital tract: a systematic review and meta-analysis. Sex Transm Dis 2008; 35:946–59. 18685546 10.1097/OLQ.0b013e3181812d15

[12] Freeman EE, Orroth KK, White RG et al Proportion of new HIV infections attributable to herpes simplex 2 increases over time: simulations of the changing role of sexually transmitted infections in sub-Saharan African HIV epidemics. Sex Transm Infect 2007; 83(Suppl 1):i17–24. 17405782 10.1136/sti.2006.023549

[13] White RG, Orroth KK, Glynn JR et al Treating curable sexually transmitted infections to prevent HIV in Africa: still an effective control strategy?J Acquir Immune Defic Syndr 2008; 47:346–53. 18176323 10.1097/QAI.0b013e318160d56a PMC3776949

[14] Buvé A, Caraël M, Hayes RJ et al; Study Group on Heterogeneity of HIV Epidemics in African Cities The multicentre study on factors determining the differential spread of HIV in four African cities: summary and conclusions. AIDS 2001; 15(Suppl 4):S127–31. 10.1097/00002030-200108004-00014 11686461

[15] Orroth KK, Freeman EE, Bakker R et al Understanding the differences between contrasting HIV epidemics in east and west Africa: results from a simulation model of the Four Cities Study. Sex Transm Infect 2007; 83(Suppl 1):i5–16. 17405781 10.1136/sti.2006.023531

[16] Zhu J, Hladik F, Woodward A et al Persistence of HIV-1 receptor-positive cells after HSV-2 reactivation is a potential mechanism for increased HIV-1 acquisition. Nat Med 2009; 15:886–92. 19648930 10.1038/nm.2006 PMC2723183

[17] Johnson KE, Redd AD, Quinn TC et al Effects of HIV-1 and herpes simplex virus type 2 infection on lymphocyte and dendritic cell density in adult foreskins from Rakai, Uganda. J Infect Dis 2011; 203:602–9. 21220779 10.1093/infdis/jiq091 PMC3071278

[18] Watson-Jones D, Weiss HA, Rusizoka M et al; HSV trial team; Steering and Data Monitoring Committees Effect of herpes simplex suppression on incidence of HIV among women in Tanzania. N Engl J Med 2008; 358:1560–71. 18337596 10.1056/NEJMoa0800260 PMC2643126

[19] Celum C, Wald A, Hughes J et al; HPTN 039 Protocol Team Effect of aciclovir on HIV-1 acquisition in herpes simplex virus 2 seropositive women and men who have sex with men: a randomised, double-blind, placebo-controlled trial. Lancet 2008; 371:2109–19. 18572080 10.1016/S0140-6736(08)60920-4 PMC2650104

[20] Celum C, Wald A, Lingappa JR et al; Partners in Prevention HSV/HIV Transmission Study Team Acyclovir and transmission of HIV-1 from persons infected with HIV-1 and HSV-2. N Engl J Med 2010; 362:427–39. 20089951 10.1056/NEJMoa0904849 PMC2838503

[21] Freeman EE, White RG, Bakker R et al Population-level effect of potential HSV2 prophylactic vaccines on HIV incidence in sub-Saharan Africa. Vaccine 2009; 27:940–6. 19071187 10.1016/j.vaccine.2008.11.074 PMC2686080

[22] Belshe RB, Leone PA, Bernstein DI et al; Herpevac Trial for Women Efficacy results of a trial of a herpes simplex vaccine. N Engl J Med 2012; 366:34–43. 22216840 10.1056/NEJMoa1103151 PMC3287348

[23] Bernstein DI, Wald A, Warren T et al Therapeutic Vaccine for Genital Herpes Simplex Virus-2 Infection: Findings From a Randomized Trial. J Infect Dis 2017; 215:856–64. 28329211 10.1093/infdis/jix004 PMC7206854

[24] Gottlieb SL, Deal CD, Giersing B et al The global roadmap for advancing development of vaccines against sexually transmitted infections: Update and next steps. Vaccine 2016; 34:2939–47. 27105564 10.1016/j.vaccine.2016.03.111 PMC6759054

[25] Hayes R, Floyd S, Schaap A et al; HPTN 071 (PopART) Study Team A universal testing and treatment intervention to improve HIV control: One-year results from intervention communities in Zambia in the HPTN 071 (PopART) cluster-randomised trial. PLoS Med 2017; 14:e1002292. 28464041 10.1371/journal.pmed.1002292 PMC5412988

[26] Patel EU, Manucci J, Kahle EM et al Precision of the Kalon Herpes Simplex Virus Type 2 IgG ELISA: an international inter-laboratory assessment. BMC Infect Dis 2015; 15:398. 26423888 10.1186/s12879-015-1130-6 PMC4591065

[27] Victora CG, Huttly SR, Fuchs SC, Olinto MT The role of conceptual frameworks in epidemiological analysis: a hierarchical approach. Int J Epidemiol 1997; 26:224–7. 9126524 10.1093/ije/26.1.224

[28] Looker KJ, Elmes JAR, Gottlieb SL et al Effect of HSV-2 infection on subsequent HIV acquisition: an updated systematic review and meta-analysis. Lancet Infect Dis 2017; 17:1303–16. 28843576 10.1016/S1473-3099(17)30405-X PMC5700807

[29] Omori R, Nagelkerke N, Abu-Raddad LJ HIV and herpes simplex virus type 2 epidemiological synergy: misguided observational evidence? A modelling study. Sex Transm Infect 2017. doi: 10.1136/sextrans-2017-053336. 10.1136/sextrans-2017-053336 PMC6204970 29203577

