# Supplementary figures and images for: Sexually Transmitted Bedfellows: Exquisite Association Between HIV and Herpes Simplex Virus Type 2 in 21 Communities in Southern Africa in the HIV Prevention Trials Network 071 (PopART) Study
Source: J Infect Dis. 2018 Apr 6;218(3):443–52. doi: 10.1093/infdis/jiy178 (PMC6049005; doi:10.1093/infdis/jiy178)

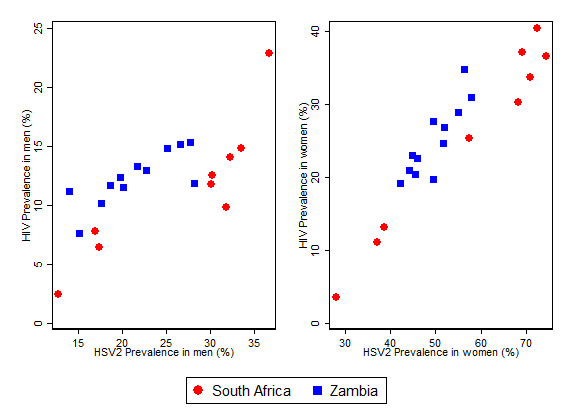

Supplement: Supplementary Figure S1 [file jiy178_suppl_supplementary_fig_s1.png]

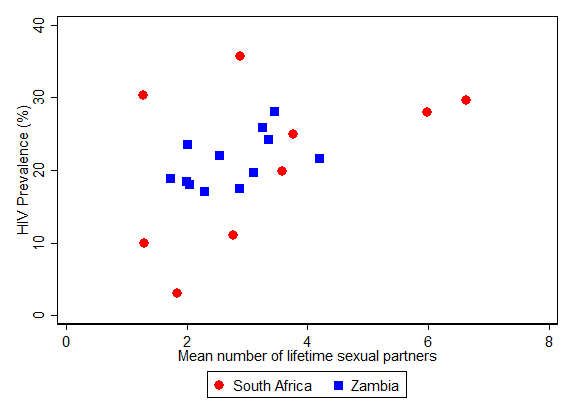

Supplement: Supplementary Figure S2 [file jiy178_suppl_supplementary_fig_s2.png]
