# Supplementary material for: Sexually Transmitted Bedfellows: Exquisite Association Between HIV and Herpes Simplex Virus Type 2 in 21 Communities in Southern Africa in the HIV Prevention Trials Network 071 (PopART) Study
Source: J Infect Dis. 2018 Apr 6;218(3):443–52. doi: 10.1093/infdis/jiy178 (PMC6049005; doi:10.1093/infdis/jiy178)
Supplement: Supplementary Table s1 [file jiy178_suppl_supplementary_table_s1.docx]

**Table S1: HSV2 prevalence by age, sex and country**

| Age | South Africa | | Zambia | |
| --- | --- | --- | --- | --- |
|  | Women | Men | Women | Men |
|  | HSV2+/Total (%) | HSV2+/Total (%) | HSV2+/Total (%) | HSV2+/Total (%) |
| 18 | 109/541 (20%) | 15/311 (5%) | 121/902 (13%) | 15/408 (4%) |
| 19 | 147/539 (27%) | 19/294 (6%) | 191/1057 (18%) | 18/516 (3%) |
| 20 | 150/510 (29%) | 18/340 (5%) | 247/908 (27%) | 19/457 (4%) |
| 21 | 212/556 (38%) | 18/314 (6%) | 295/907 (33%) | 30/383 (8%) |
| 22 | 268/589 (46%) | 38/301 (13%) | 309/861 (36%) | 30/346 (9%) |
| 23 | 263/579 (45%) | 34/292 (12%) | 309/761 (41%) | 34/300 (11%) |
| 24 | 325/580 (56%) | 49/266 (18%) | 283/639 (44%) | 35/247 (14%) |
| 18-24 | 1474/3894 (38%) | 191/2118 (9%) | 1755/6035 (29%) | 181/2657 (7%) |
| 25-29 | 1749/2778 (63%) | 295/1191 (25%) | 1657/3034 (55%) | 209/957 (22%) |
| 30-34 | 1648/2317 (71%) | 364/894 (41%) | 1539/2335 (66%) | 264/711 (37%) |
| 35-39 | 1356/1761 (77%) | 320/669 (48%) | 1192/1574 (76%) | 279/556 (50%) |
| 40-44 | 1025/1427 (72%) | 304/600 (51%) | 810/1022 (79%) | 223/427 (52%) |
| Total | 7253/12179 (60%) | 1474/5472 (27%) | 6954/14002 (50%) | 1156/5309 (22%) |
