# Supplementary material for: Sexually Transmitted Bedfellows: Exquisite Association Between HIV and Herpes Simplex Virus Type 2 in 21 Communities in Southern Africa in the HIV Prevention Trials Network 071 (PopART) Study
Source: J Infect Dis. 2018 Apr 6;218(3):443–52. doi: 10.1093/infdis/jiy178 (PMC6049005; doi:10.1093/infdis/jiy178)
Supplement: Supplementary Table s2 [file jiy178_suppl_supplementary_table_s2.docx]

**Table S2: Risk factors for HSV2 infection in South Africa**

|  | **Women** | | | **Men** | | |
| --- | --- | --- | --- | --- | --- | --- |
| **Variable** | HSV2+/Total (%) | OR^1^ (95% CI) | Adj OR^2^ (95% CI) | HSV2+/Total (%) | OR^1^ (95% CI) | Adj OR^2^ (95% CI) |
| Education  None/Grade 1-2  Grade 3-6  Grade 7-10  Grade 11-12  College/University |  | P < 0.001 | P < 0.001 |  | P = 0.8277 | P = 0.4602 |
|  | 40/60 (40%) | 1 | 1 | 17/50 (34%) | 1 | 1 |
|  | 274/388 (71%) | 1.13 (0.69-2.48) | 0.91 (0.38-2.19) | 88/255 (35%) | 1.00 (0.50-2.00) | 1.05 (0.47-2.31) |
|  | 2,548/4,158 (61%) | 0.91 (0.50-1.65) | 0.64 (0.28-1.45) | 507/1,917 (26%) | 1.11 (0.58-2.11) | 1.39 (0.67-2.88) |
|  | 4,060/6,869 (59%) | 0.74 (0.41-1.34) | 0.55 (0.24-1.26) | 764/2,851 (27%) | 1.13 (0.59-2.14) | 1.42 (0.69-2.95) |
|  | 244/539 (45%) | 0.42 (0.23-0.78) | 0.36 (0.15-0.84) | 61/275 (22%) | 0.97 (0.48-1.96) | 1.25 (0.55-2.84) |
| Marital Status |  | P < 0.001 | P < 0.001 |  | P = 0.011 | P = 0.145 |
| Married | 2,307/3,622 (64%) | 1 | 1 | 377/927 (41%) | 1 | 1 |
| Never married | 4,641/8,137 (57%) | 1.47 (1.34-1.62) | 1.37 (1.21-1.55) | 1,023/4,410 (23%) | 1.09 (0.91-1.29) | 1.17 (0.92-1.49) |
| Divorced/separated | 220/299 (74%) | 1.44 (1.08-1.92) | 1.28 (0.85-1.93) | 61/97 (63%) | 2.03 (1.30-3.19) | 2.02 (1.05-3.88) |
| Widowed | 64/83 (77%) | 1.38 (0.80-2.38) | 1.95 (0.71-5.34) | 8/12 (67%) | 2.34 (0.67-8.14) | 0.81 (0.17-3.92) |
| Circumcision | - | - | - |  | P < 0.001 | P < 0.001 |
| Not circumcised | - | - | - | 310/1,912 (16%) | 1 | 1 |
| Voluntary medical male circumcision (VMMC) | - | - | - | 92/455 (20%) | 0.96 (0.72-1.27) | 1.01 (0.69-1.46) |
| Traditional male circumcision (TMC) | - | - | - | 1,012/2,844 (36%) | 1.87 (1.54-2.26) | 1.70 (1.31-2.21) |

^1^ Adjusted for age-group and community ^2^ Adjusted for age-group, community, lifetime sexual partners, audit score, recreational drug use, education, marital status, nights away from home in last 3 months, SES, condom use, age at first sex, and, for men, circumcision.
