# Supplementary material for: Sexually Transmitted Bedfellows: Exquisite Association Between HIV and Herpes Simplex Virus Type 2 in 21 Communities in Southern Africa in the HIV Prevention Trials Network 071 (PopART) Study
Source: J Infect Dis. 2018 Apr 6;218(3):443–52. doi: 10.1093/infdis/jiy178 (PMC6049005; doi:10.1093/infdis/jiy178)
Supplement: Supplementary Table s3 [file jiy178_suppl_supplementary_table_s3.docx]

**Table S3: Risk factors for HSV2 infection in Zambia**

|  | **Women** | | | **Men** | | |
| --- | --- | --- | --- | --- | --- | --- |
| **Variable** | HSV2+/Total (%) | OR^1^ (95% CI) | Adj OR^2^ (95% CI) | HSV2+/Total (%) | OR^1^ (95% CI) | Adj OR^2^ (95% CI) |
| Education  None/Grade 1-2  Grade 3-6  Grade 7-10  Grade 11-12  College/University |  | P < 0.001 | P < 0.001 |  | P < 0.001 | P = 0.003 |
|  | 309/511 (60%) | 1 | 1 | 27/62 (44%) | 1 | 1 |
|  | 1,055/1,732 (61%) | 1.16 (0.93-1.44) | 1.29 (0.98-1.70) | 102/275 (37%) | 0.79 (0.43-1.45) | 0.46 (0.20-1.07) |
|  | 4,013/7,382 (54%) | 1.06 (0.86-1.29) | 1.21 (0.94-1.57) | 597/2,211 (27%) | 0.79 (0.45-1.38) | 0.52 (0.24-1.14) |
|  | 1,217/3,548 (34%) | 0.58 (0.47-0.72) | 0.88 (0.67-1.16) | 326/2,250 (15%) | 0.53 (0.30-0.94) | 0.44 (0.20-0.96) |
|  | 331/780 (42%) | 0.52 (0.40-0.67) | 0.66 (0.47-0.92) | 97/491 (20%) | 0.40 (0.22-0.73) | 0.28 (0.12-0.64) |
| Marital Status |  | P < 0.001 | P < 0.001 |  | P < 0.001 | P < 0.001 |
| Married | 4673/8,563 (55%) | 1 | 1 | 698/1,752 (40%) | 1 | 1 |
| Never married | 827/3,499 (24%) | 0.47 (0.42-0.52) | 0.66 (0.56-0.77) | 268/3,109 (9%) | 0.41 (0.33-0.50) | 0.40 (0.30-0.53) |
| Divorced/separated | 1,033/1,430 (72%) | 2.02 (1.78-2.30) | 1.44 (1.19-1.75) | 157/387 (41%) | 0.99 (0.78-1.25) | 0.67 (0.47-0.96) |
| Widowed | 399/457 (87%) | 3.19 (2.39-4.25) | 3.31 (1.85-5.90) | 26/45 (58%) | 1.33 (0.72-2.46) | 1.00 (0.33-3.01) |
| Circumcision |  |  |  |  | P = 0.126 | P = 0.780 |
| Not circumcised | - | - | - | 859/3,562 (24%) | 1 | 1 |
| Voluntary medical male circumcision (VMMC) | - | - | - | 201/1,356 (15%) | 0.82 (0.68-0.99) | 0.94 (0.76-1.17) |
| Traditional male circumcision (TMC) | - | - | - | 67/258 (26%) | 0.99 (0.72-1.36) | 0.91 (0.63-1.30) |

^1^ Adjusted for age-group and community ^2^ Adjusted for age-group, community, lifetime sexual partners, audit score, recreational drug use, education, marital status, nights away from home in last 3 months, SES, condom use, age at first sex, and, for men, circumcision.
