# Supplementary material for: Sexually Transmitted Bedfellows: Exquisite Association Between HIV and Herpes Simplex Virus Type 2 in 21 Communities in Southern Africa in the HIV Prevention Trials Network 071 (PopART) Study
Source: J Infect Dis. 2018 Apr 6;218(3):443–52. doi: 10.1093/infdis/jiy178 (PMC6049005; doi:10.1093/infdis/jiy178)
Supplement: Supplementary Table s4 [file jiy178_suppl_supplementary_table_s4.docx]

**Table S4: HSV2 prevalence by community**

| Country | Community | HSV2+/Total (%) in women | HSV2+/Total (%) in men | HIV+/Total (%) in women | HIV+/Total (%) in men |
| --- | --- | --- | --- | --- | --- |
| South Africa | A | 242/862 (28%) | 79/618 (13%) | 30/868 (4%) | 15/620 (2%) |
| South Africa | B | 502/1351 (37%) | 107/630 (17%) | 150/1351 (11%) | 49/630 (8%) |
| South Africa | C | 445/1149 (39%) | 90/517 (17%) | 151/1149 (13%) | 33/518 (6%) |
| South Africa | D | 874/1520 (58%) | 242/761 (32%) | 386/1526 (25%) | 75/762 (10%) |
| South Africa | E | 961/1409 (68%) | 180/595 (30%) | 427/1412 (30%) | 75/598 (13%) |
| South Africa | F | 1104/1451 (69%) | 225.671 (34%) | 514/1459 (37%) | 100/675 (15%) |
| South Africa | G | 924/1304 (71%) | 172/533 (32%) | 440/1307 (34%) | 75/533 (14%) |
| South Africa | H | 1043/1442 (72%) | 189/516 (37%) | 585/1447 (40%) | 118/516 (23%) |
| South Africa | I | 1258/1691 (74%) | 190/631 (30%) | 620/1695 (37%) | 74/630 (12%) |
| Zambia | J | 315/746 (42%) | 39/278 (15%) | 143/746 (19%) | 31/278 (11%) |
| Zambia | K | 563/1273 (44%) | 83/420 (20%) | 267/1273 (21%) | 52/420 (12%) |
| Zambia | L | 228/641 (45%) | 52/343 (15%) | 147/641 (23%) | 26/343 (8%) |
| Zambia | M | 551/1212 (45%) | 90/415 (22%) | 247/1213 (20%) | 55/415 (13%) |
| Zambia | N | 707/1535 (46%) | 105/564 (19%) | 346/1535 (23%) | 66/565 (12%) |
| Zambia | O | 771/1561 (49%) | 114/405 (28%) | 308/1561 (20%) | 48/405 (12%) |
| Zambia | P | 599/1212 (49%) | 99/563 (18%) | 336/1213 (28%) | 57/563 (10%) |
| Zambia | Q | 556/1074 (52%) | 63/313 (20%) | 265/1074 (25%) | 36/313 (12%) |
| Zambia | R | 912/1753 (52%) | 161/641 (25%) | 470/1754 (27%) | 95/641 (15%) |
| Zambia | S | 609/1106 (55%) | 102/449 (23%) | 319/1106 (295) | 58/449 (13%) |
| Zambia | T | 390/692 (56%) | 99/358 (28%) | 241/692 (35%) | 55/358 (15%) |
| Zambia | U | 693/1197 (58%) | 149/560 (27%) | 371/1197 (31%) | 85/560 (15%) |
