# Supplementary material for: Sexually Transmitted Bedfellows: Exquisite Association Between HIV and Herpes Simplex Virus Type 2 in 21 Communities in Southern Africa in the HIV Prevention Trials Network 071 (PopART) Study
Source: J Infect Dis. 2018 Apr 6;218(3):443–52. doi: 10.1093/infdis/jiy178 (PMC6049005; doi:10.1093/infdis/jiy178)
Supplement: Supplementary Table s5 [file jiy178_suppl_supplementary_table_s5.docx]

**Table S5: Linear association between cluster level HIV prevalence and cluster level HSV2 prevalence and cluster level mean lifetime sexual partners**

| Factor | Effect on HIV prevalence % for a 1 unit increase | Adjusted^1^ effect on HIV prevalence % for a 1 unit increase |
| --- | --- | --- |
| Cluster level HSV2 prevalence (%) | 0.62 (0.49-0.75)  p < 0.001 | 0.67 (0.51-0.83)  p < 0.001 |
| Cluster level mean lifetime sexual partners | 2.51 (0.15-4.86)  p = 0.038 | -0.61 (-1.90-0.68)  p = 0.336 |

^1^ Adjusted for other factor in table
